# Expression of annexin A2 in adenomyosis and dysmenorrhea

**DOI:** 10.1007/s00404-019-05205-w

**Published:** 2019-06-10

**Authors:** Feng Liu, Lixue Liu, Jian Zheng

**Affiliations:** Department of Gynecology and Obstetrics, The Affiliated Hospital of Inner Mongolia Medical University, Huhhot, People’s Republic of China

**Keywords:** Annexin A2, Adenomyosis, Dysmenorrhea

## Abstract

**Purpose:**

To investigate the expression of annexin A2 (ANXA2) in ectopic and eutopic endometrium and serum of women with adenomyosis, and their relationships with adenomyosis-associated dysmenorrhea.

**Methods:**

The expression of ANXA2 was detected by immunohistochemical S-P method in ectopic and eutopic endometrium tissues from 30 patients with adenomyosis who underwent hysterectomy. The correlation between ANXA2 expression and dysmenorrhea degree was evaluated. The endometrium tissues from 15 patients with uterine fibroids which underwent hysterectomy were used as controls. The preoperative serum level of ANXA2 was measured by enzyme-linked immunosorbent assay in 30 patients with adenomyosis and 15 patients with hysteromyoma.

**Result:**

The expression of ANXA2 in eutopic and ectopic endometrium of adenomyosis was higher than in normal endometrium (*P* < 0.05), but no significant difference of ANXA2 expression was observed between the eutopic endometrium and the ectopic endometrium (*P* > 0.05). In the ectopic endometrium, but not in the eutopic endometrium, of women with adenomyosis, ANXA2 expression was positively correlated with the severity of dysmenorrhea (*R* = 0.831, *P* = 0.000). The preoperative serum level of ANXA2 was markedly higher in patients with adenomyosis compared with the patients with hysteromyoma (*P* < 0.05).

**Conclusion:**

The increased ANXA2 may contribute to the occurrence and development of adenomyosis, and may play a important role in the dysmenorrhea. The present study may provide a new idea of diagnosis and treatment to adenomyosis-associated dysmenorrhea.

## Introduction

Adenomyosis is a benign gynecological disease characterized by the presence of aberrant growth and invasion of endometrial tissue embedded within the myometrium, leading to dysfunctional myometrial hyperperistalsis, increased intra-uterine pressure and impairment of proper uterine function [1, 2]. Detection of serum CA125 is good for diagnosis, but definitive diagnosis still requires surgery and pathology. Due to the lack of reliable diagnostic indicators, it is inevitable to cause misdiagnosis or missed diagnosis. Therefore, it is of profound significance to find a diagnostic marker. In addition, moderate and severe dysmenorrhea seriously affects the physical and mental health and quality of life of women of childbearing age, and the relevant mechanism is not yet very clear, the root cause of urgent need to investigate. Recent studies have found that epithelial–mesenchymal transition (EMT) is the initiating factor of AM [3, 4]. The in vitro AM model confirmed that estrogen significantly up-regulated ANXA2 and induced EMT [5]. Additionally, evidence-based data unraveled an active role for ANXA2 in the pathogenesis of adenomyosis through conferring the ability of endometrial carcinomas to metastasize and proangiogenic capacity [6]. Annexin A2 (ANXA2), a calcium-binding cytoskeletal protein found on various cell types, has a diverse range of cellular functions including angiogenesis, proliferation, apoptosis, calcium signaling and cell growth regulation [7–9]. The up-regulated expression of ANXA2 has been reported in breast cancer, pancreatic cancer and laryngeal cancer tissue [10]. However, little is known about the relationship between ANXA2 and adenomyosis-associated dysmenorrhea. In this study, the expression of ANXA2 was detected by immunohistochemical S-P method, followed by the Pearson correlations for the correlation analysis of ANXA2 with adenomyosis-associated dysmenorrhea. Meanwhile, the levels of preoperative serum ANXA2 of patients with adenomyosis (*n* = 30) and uterine myoma (*n* = 15) were also measured by enzyme-linked immunosorbent assay (ELISA). These findings are to provide a reliable theoretical basis for the clinical diagnosis and treatment of adenomyosis-associated dysmenorrhea and the development and application of related markers.

## Materials and methods

### Tissue collection

Freshly resected matched ectopic and eutopic endometrial tissues of adenomyosis and adenomyoma patients undergoing hysterectomy were collected from the department of gynecology and obstetrics, the affiliated hospital of Inner Mongolia medical university from January 2018 to December 2018. None of pre-menopausal had intra-uterine device (IUD), typical endometrial hyperplasia; none unincorporated endocrine, immune and metabolic disease, malignant tumors, and none received any hormone and immune agents prior to surgery for 3 months. Tissue and serum samples were immediately frozen in liquid nitrogen and stored at − 80 °C.

### Immunohistochemistry

*Experimental group*: ectopic and eutopic endometrial tissues of 30 donors with an average of 42 years old (31–48 years old) were documented by pathology department. There were 15 cases of proliferative phase and secretory phase in 15 cases with a history of uterine cavity operation no more than 2 times (diagnostic curettage or abortion, etc.), respectively. Of these, there were ten cases of mild dysmenorrhea, ten cases of moderate dysmenorrhea, and ten cases of severe dysmenorrhea.

*Control group*: 15 normal endometrium tissues were obtained from adenomyoma patients with an average of 40 years old (33–50 years old).

4-μm tissue sections were set in oven at 60 °C for 20 min, and then xylene was used to dewaxing twice, 10 min each time. Tissue sections were incubated with 100% alcohol for 10 min twice, 95, 90 and 80% alcohol for 5 min, respectively, to block endogenous peroxidase activity. After washing, sections were incubated with a repairing solution for 9 min. 50 μL ANXA2 antibody (Bio-synthesis, Lewisville, USA) 1:100 was diluted in blocking solution and incubated in a humidified chamber at 4 °C for one night. After washing, 50 μL secondary antibody (biotin-labeled goat anti-mouse IgG) was applied for 10 min at room temperature followed by 3 washing steps of 3 min each, and then 50 μL streptavidin peroxidase solution was used. Sections were then washed, and color was developed using DAB substrate chromogen system (Zymed Inc., South San Francisco, CA, USA). Sections were counterstained with Mayer hematoxylin for 3 min. Finally, sections were analyzed with a microscope (BH-2 OLYMPUS, Tokyo, Japan).

### ELISA

*Experimental group*: Preoperative serum samples were obtained from 30 adenomyosis patients, with an average of 42 years (ranging from 31 to 48 years old).

*Control group*: preoperative serum samples of 15 patients with uterine myoma ranged from 33 to 50 years old, with an average of 40 years.

The level of serum ANXA2 was detected using a human ANXA2 ELISA kit (Uscn Life Science Inc, Wuhan, China) according to the manufacturer’s instructions. 50 μL of serum sample or standard separately was added to each well, and then 50 μL of detection reagent was applied and incubated for 30 min at 37 °C. Subsequently, 50 μL color development reagent A and 50 μL reagent B were added and incubated for 15 min at 37 °C. Finally, 50 μL of stop solution was added to each well, and absorbance was read at 450 nm. During the procedure, washing the plate was according to the ELISA routine method.

### Evaluation criteria

*Staining and scoring*: ANXA2 exhibited positive expression with yellow or brown particles in membrane and cytoplasm. To evaluate the immunostaining, a score corresponding to the product of both (a) staining (0 = negative; 1 = canary; 2 = yellow; 3 = brown) and (b) percentage of positive cells (0 = < 5% positive cells; 1 = 5–25% positive cells; 2 = 26–50% positive cells; 3 = 51–75% positive cells; 4 ≥ 75%) was established. The product of (a) × (b) was considered as comprehensive evaluation scores (0–2 = negative; 3–4 = weakly positive; 5–8 = positive; 9–12 = strongly positive). A score greater than 2 was the value of a positive immunohistochemical assay [11, 12]. The results of staining were evaluated by two independent pathologists without knowledge of the clinico-pathological features, and any difference in interpretation was resolved by consensus.

*Dysmenorrhea degree*: The examiners evaluated the degree of dysmenorrhea using a visual analog scale [13]. (VAS, ranged 0–10 cm, a score of “10” entailed the best outcome, while a score of “0” entailed the worst). Pain assessment criteria: no dysmenorrhea (−), mild pain (+, l–4 cm), moderate pain (++ , 5–7 cm) and severe pain (+++, 8–l0 cm).

### Statistical analysis

IBM SPSS statistical software, version 13 for Windows (IBM SPSS Inc., Chicago, IL, USA) was used for the statistical analysis. The experimental data were shown as mean ± SD. One-way ANOVA was used with Tukey’s multiple comparison tests for multiple groups. Bivariate Pearson correlation was used for the correlation analysis. The level of statistical significance was set at 0.05.

## Results

### ANXA2 antibody staining

**Fig. 1:**
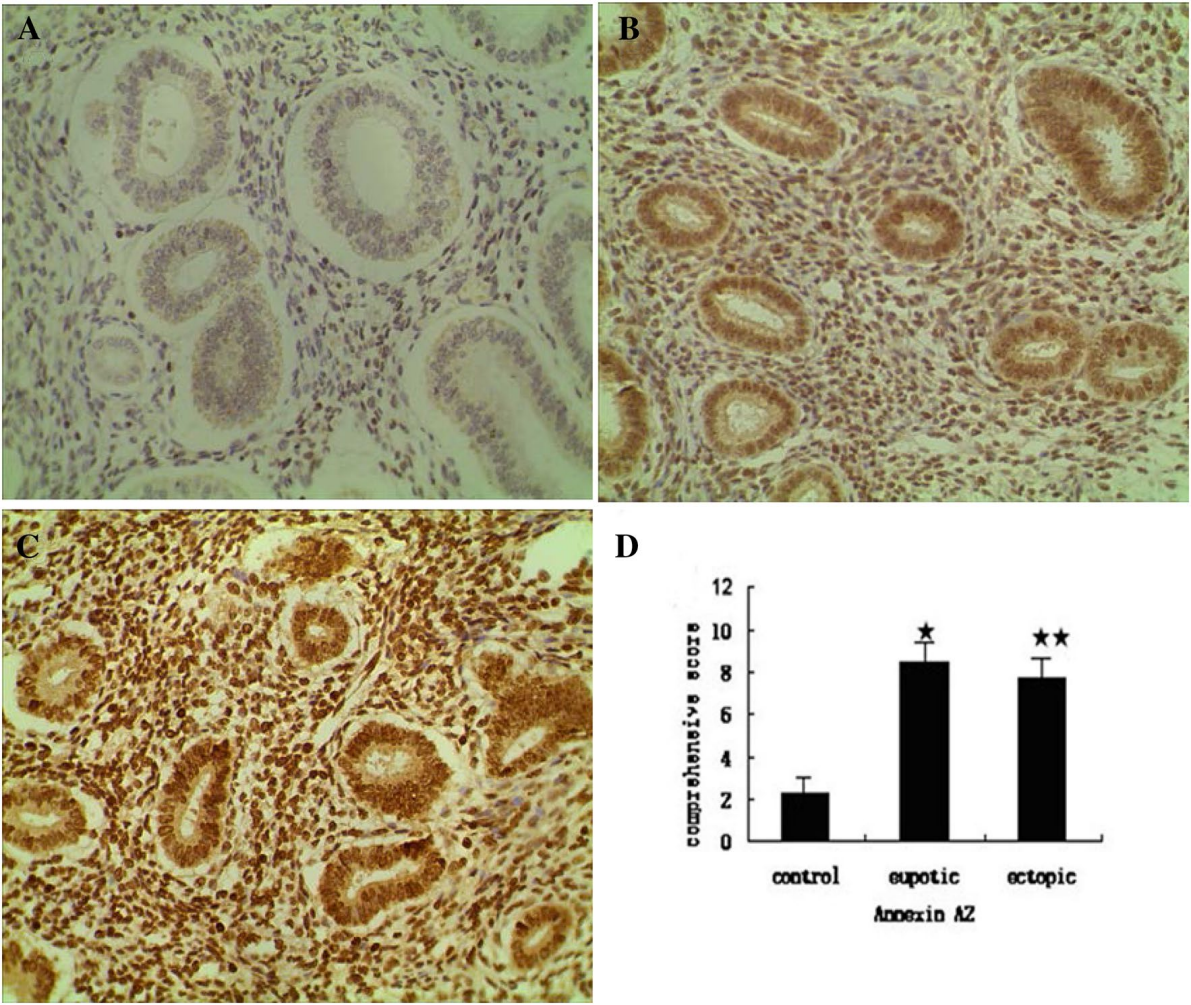
The expression of ANXA2 in controls, eutopic endometrium and ectopic endometrium. **a** ANXA2 was weakly positively expressed in normal endometrium tissues. **b** ANXA2 was strongly positively expressed in eutopic endometrium. **c** ANXA2 was positively expressed in ectopic endometrium. **d** Semi-quantitatively for ANXA2 of three groups (*P < 0.05, **P < 0.01) compared with control group  × 400

According to the immunohistochemistry (Fig. 1a–c) and the comprehensive scoring criteria, ANXA2 was strongly positive expressed in eutopic endometrium, and was positively expressed in ectopic endometrium. The result of semi-quantitatively for ANXA2 showed that there was a significant difference between control group and adenomyosis groups (Fig. 1d). ANXA2 was significantly increased in adenomyosis. However, the expression of ANXA2 had no significant difference between eutopic endometrium group and ectopic endometrium group.

### ANXA2 expression and dysmenorrhea degree in adenomyosis groups

**Fig. 2:**
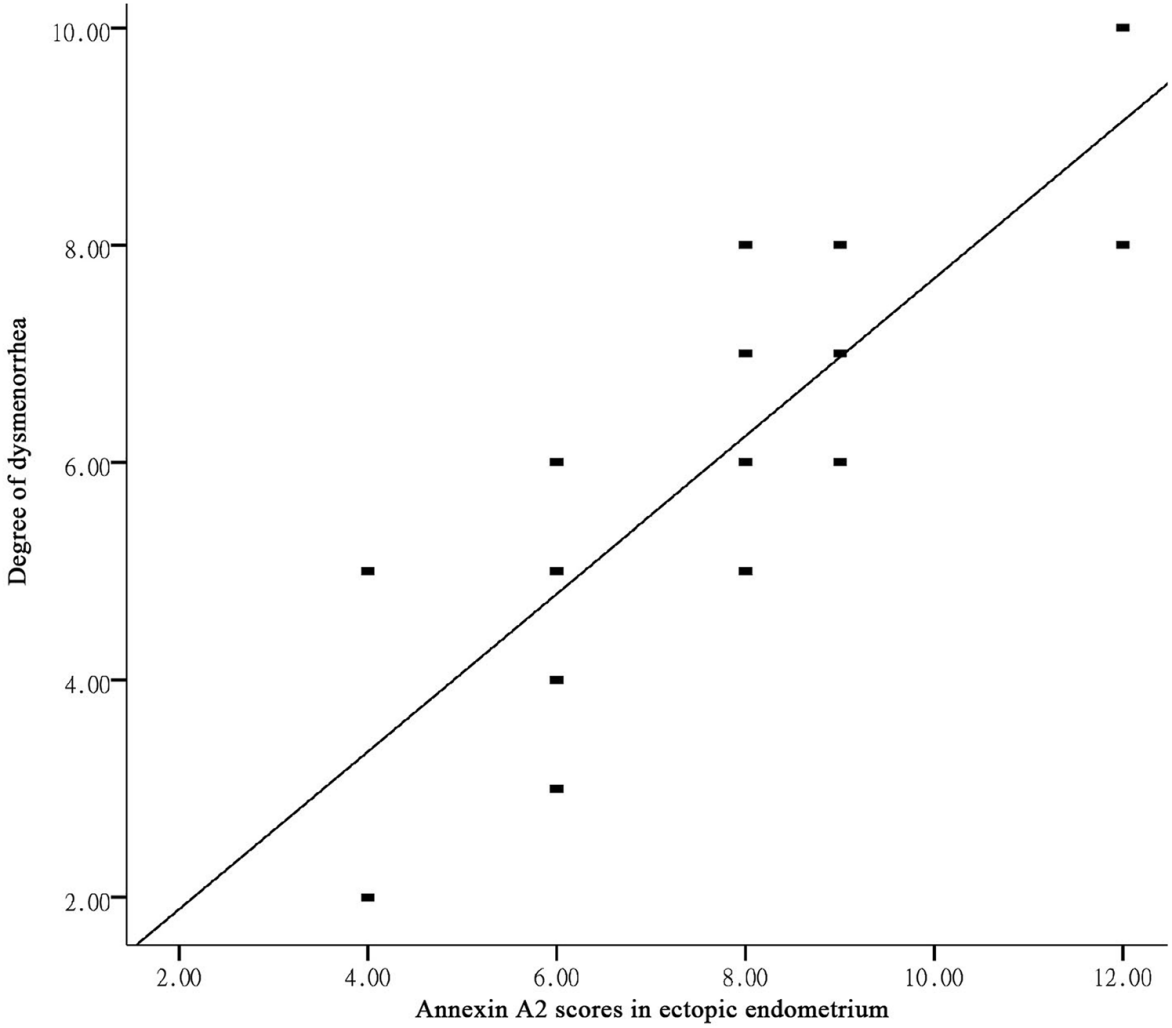
ANXA2 expression was positively correlated with dysmenorrhea degree in ectopic endometrium

As shown in Fig. 1, ANXA2 was significantly increased in eutopic endometrium and ectopic endometrium. Further, to investigate the correlation between ANXA2 expression and dysmenorrhea degree in eutopic endometrium and ectopic endometrium, Pearson correlations analysis was performed. The result revealed that there was a significant positive correlation between ANXA2 expression in ectopic endometrium and the degree of dysmenorrhea (*R* = 0.831, *P* = 0.000, Fig. 2), however, ANXA2 expression in eutopic endometrium had no obviously linear dependence on dysmenorrheal degree (*R* = 0.187, *P *= 0.121).

### Level of ANXA2 in serum of patients with adenomyosis

**Fig. 3:**
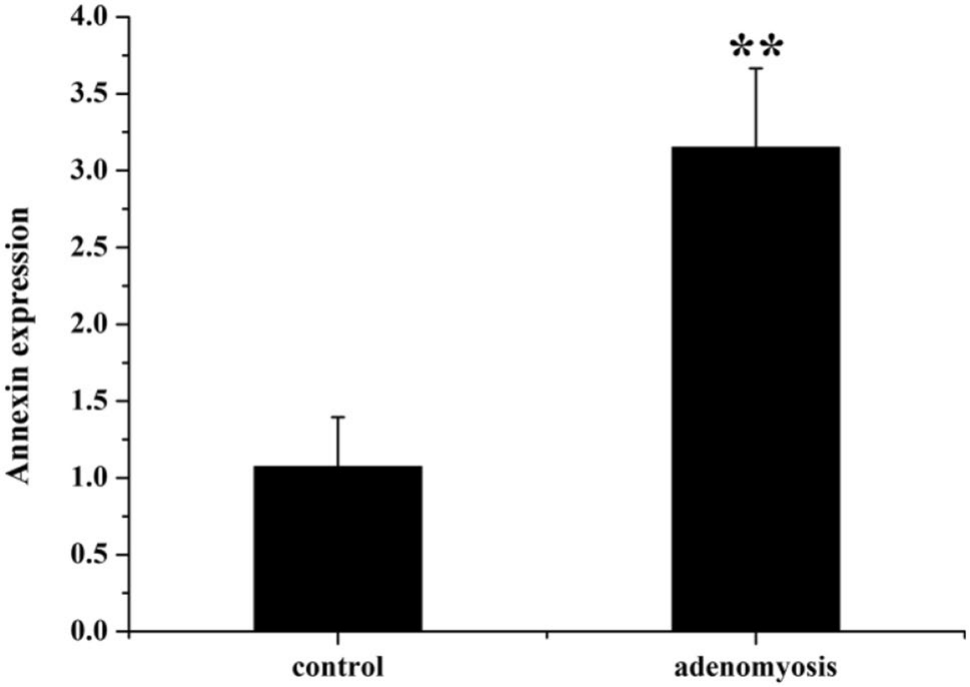
The ANXA2 level in preoperative serum of control and adenomyosis groups. (**P < 0.01, compared with control group)

The ANXA2 level in preoperative serum was further detected by ELISA (Fig. 3). Serum level of ANXA2 in adenomyosis group was significant higher than that in control group (*P* < 0.01).

## Discussion

Multiple pregnancies, childbirth, induced abortion, chronic endometritis and other factors make the endometrial basal layer thinning and loss of protective function. The endometrium grows through direct contact with the myometrium through the unprotected areas and invades the myometrium, and further invades the surrounding tissue. In the observation of pathological sections of AM patients undergoing hysterectomy, endometrial glands and stromal tissues were found in 10–47% of the myometrium, which were closely connected with the endometrium. Therefore, although AM is a benign pathological manifestation, it has biological characteristics similar to malignant tumors, such as implantation, growth, infiltration, recurrence and so on. In addition to the basement membrane invagination theory, the pathogenesis of AM may be related to estrogen metabolism disorder, EMT, eutopic endometrial lesion, immune factors and genetic factors. From the perspective of EMT, exploring the pathogenesis of AM will become an important research direction in the future. Research finds promotion for the growth, distant metastasis and angiogenesis of AM endometrial tissue is implemented through the mechanism on ANXA2-inducing EMT [5]. ANXA2 was first discovered in 1979 by Rade and Martin. Its basic structure contains 339 amino acids, consisting of the N-terminal of 3 kD and the C-terminal domain of 33 kD. As a calcium-binding cytoskeleton protein, it has many functions including angiogenesis, proliferation, apoptosis, calcium signal transduction and cell growth regulation [7–9]. The mechanism on induction EMT in AM may be through binding fibrinogen then hydrolyzing to fibrinolysis enzyme, which can degrade extracellular matrix and peripheral vascular basement membrane [14]. It is possible to change cell-to-cell and cell-to-matrix adhesion by binding to cell surface adhesion molecules to enhance the anti-apoptosis and motility of cells, and then induce epithelial-to-mesenchymal cell transformation (EMT) [3, 4]. Research finds that ANXA2 abnormal expression in cervical cancer, ovarian cancer, choriocarcinoma and other gynecological malignancies [15] and AM also has biological behavior similar to a malignant tumor. According to ANXA2 function and the above in vitro study AM, the mechanism is speculated. In the human body of AM, that ANXA2 abnormal expression probably promoted the occurrence and development of AM have not been reported. In this study, the expression of ANXA2 in AM was detected by immunohistochemistry S-P method. There was no significant difference between the two groups (*P* > 0.05), but the expression was higher in eutopic endometrium and ectopic endometrium than in normal endometrium (*P* < 0.05). In vitro studies found that the increased expression about ANXA2 in AM ectopic lesions was consistent [5]. This not only confirms the abnormal expression of ANXA2 in human AM tissues, but also indicates that the effect of ANXA2 in AM epitope and ectopic endometrium may be the same, and may be different from that in uterine leiomyoma. It indicates that ANXA2 may play an important role in the development of AM.

Progressive dysmenorrhea is the main clinical manifestation of AM, seriously affecting the quality of life of patients, some patients just because of dysmenorrhea can not bear the reluctance to remove the uterus. It is reported that the dysmenorrhea rate of AM patients is as high as 64.8–77.8% [16]. It is noted that dysmenorrhea is closely related not only to estrogen but also to prostaglandins and cyclooxygenases 2. A study confirmed that estrogen significantly up-regulates ANXA2 [5]. Another study found that prostaglandin E2 increased significantly in uterine tissues of AM patients, especially in patients with severe dysmenorrhea. Cyclooxygenase 2 is the rate-limiting enzyme for the conversion of arachidonic acid to prostaglandins. It was observed that ANXA2 significantly increased the expression of Cyclooxygenase 2 in peritoneal macrophages of patients with endometriosis by PCR and Western-blot methods [17]. It can be seen that AM-elevated estrogen levels in patients may increase cyclooxygenase 2 by up-regulating ANXA2. There by increasing prostaglandin E2 production and promoting dysmenorrhea. EMT is strongly related to high estrogen environment. Estrogen-induced EMT is one of the important mechanisms of AM development. Among them, ANXA2 may play a key role. The relationship between ANXA2 expression and dysmenorrhea in AM tissues was tested in this study. The expression level of ANXA2 in AM ectopic endometrium was positively correlated with dysmenorrhea degree (*R* = 0.831, *P* = 0.000). The expression level of ANXA2 in AM ectopic endometrium increased gradually with the degree of dysmenorrhea. These results suggest that the exacerbation of dysmenorrhea is closely related to the up-regulation of ANXA2 expression in AM lesions, and there is a certain correlation between them. It is suggested that ANXA2 is involved in the occurrence of AM and dysmenorrhea, and promotes the development of AM and the aggravation of dysmenorrhea.

Preoperative diagnostic coincidence rate of AM by ultrasonography was only 52.9–60.5% and misdiagnosis rate was high. Preoperative diagnostic coincidence rate of MRI was 88.2%, but the high price limited the clinical application. Although the detection of serum CA125 has some reference value in the diagnosis of AM, the positive rate and the value of serum CA125 have not been reported in the literature. There is still a lack of sensitivity and specificity, so it is necessary to develop more reliable markers. In this study, the serum concentration of ANXA2 in AM patients was determined by ELISA for the first time, which was significantly higher than that in hysteromyoma patients (*P* < 0.05), suggesting that ANXA2 might be a new marker for the diagnosis of AM. Gene-targeting therapy for ANXA2, a biological target, provides a new therapeutic approach to alleviate dysmenorrhea in AM patients and to meet the conservative and fertility requirements of some patients after the open second-child policy.

## Conclusion

The increased ANXA2 may contribute to the occurrence and development of adenomyosis, and may play a important role in the dysmenorrhea. The present study may provide a new idea of diagnosis and treatment to adenomyosis-associated dysmenorrhea.
